# Association Between Early Skin‐To‐Skin Contact and Postpartum Maternal Wellbeing: A Retrospective Cross‐Sectional Study

**DOI:** 10.1002/nop2.70851

**Published:** 2026-10-16

**Authors:** Eva Hendrych Lorenzová, Denis Mainz, Alena Lochmannová, Colin R. Martin, Kateřina Ratislavová

**Affiliations:** ^1^ Department of Nursing and Midwifery, Faculty of Health Care Studies University of West Bohemia in Pilsen Pilsen Czech Republic; ^2^ Department of Rehabilitation Studies, Faculty of Health Care Studies University of West Bohemia in Pilsen Pilsen Czech Republic; ^3^ Department of Emergency Medicine, Diagnostic Disciplines and Public Health, Faculty of Health Care Studies University of West Bohemia in Pilsen Pilsen Czech Republic; ^4^ Institute of Health and Wellbeing University of Suffolk Ipswich UK

**Keywords:** birth satisfaction, maternal wellbeing, postnatal depression, postpartum care, posttraumatic stress disorder, skin‐to‐skin contact

## Abstract

**Aim:**

To identify perinatal factors associated with early maternal‐newborn skin‐to‐skin contact after birth and examine associations between contact duration, birth satisfaction and postpartum mental wellbeing.

**Design:**

A retrospective cross‐sectional survey design was used.

**Methods:**

An anonymous online survey included 985 women in the Czech Republic, recruited by non‐probability convenience sampling. Measures assessed perinatal characteristics, birth satisfaction, depressive symptoms and childbirth‐related posttraumatic stress symptoms. Chi‐square and Kruskal–Wallis tests included effect sizes and multiplicity‐adjusted post hoc comparisons. Multivariable linear and logistic models adjusted for maternal, pregnancy, birth and newborn factors.

**Results:**

Skin‐to‐skin contact duration was associated with parity, pregnancy course, mode and timing of birth, newborn health and time since birth. Women reporting no contact had the lowest birth satisfaction and highest mental‐health screening scores. Adjusted odds of positive screening for both outcomes (depressive and posttraumatic stress symptoms) were lower for > 30–120 and > 120 min of contact, but not ≤ 30 min, compared with no contact. All contact‐duration groups had higher adjusted birth satisfaction, with the largest difference for > 120 min.

**Conclusion:**

Early skin‐to‐skin contact was associated with higher birth satisfaction and more favourable maternal mental‐health screening outcomes. The design and sampling do not permit causal inference.

**Implications for the Profession:**

Clinical practice guidelines for midwives, obstetric and neonatal nurses should focus not only on initiating skin‐to‐skin contact but also on sustaining its duration and safely overcoming barriers during complicated births.

**Impact:**

By highlighting how the duration of early uninterrupted skin‐to‐skin contact is associated with long‐term maternal mental well‐being, this study contributes valuable insights to maternal and child healthcare and underscores the psychological relevance of early mother‐infant contact.

**Reporting Method:**

This study adhered to the Strengthening the Reporting of Observational Studies in Epidemiology guidelines.

**Patient or Public Contribution:**

There was no patient or public involvement; postpartum women participated as consenting survey respondents.

## 1 Introduction

Pregnancy, childbirth and the postpartum period are volatile, fragile and emotionally sensitive times for women, which can also be very distressing. Emphasizing women's perinatal mental health should be part of any holistic obstetric and nursing care. Women's experience of childbirth has been shown to influence their subsequent quality of life (Martínez‐Galiano et al. 2019).

Early skin‐to‐skin contact (SSC) between the woman and the newborn after birth is a very important part of the birth process and is referred to as evidence‐based best practice. Early, continuous and uninterrupted SSC after birth should be a standard part of obstetric care for mother and baby (from 1000 g with experienced staff if assistance is needed) in all types of births (Brimdyr et al. 2023). SSC after birth can be described as contact of the naked newborn on the mother's bare chest. It is most appropriate to encourage immediate SSC contact between mother and newborn after birth, without any interruption, allowing for self‐suckling to the breast.

Although the physiological and breastfeeding benefits of SSC are well documented, evidence on associations between SSC duration, maternal birth satisfaction and postpartum mental wellbeing remains limited, particularly beyond the immediate postnatal period.

## 2 Background

Research has found that early SSC between the postpartum woman and the newborn increases women's satisfaction with the birth experience (Kahalon et al. 2021; Mazúchová et al. 2020). Women's experience of obstetric care can have a significant impact on their future somatic and psychological health. A positive experience of childbirth can support women in their personal development and positive self‐perception, helping women to be more satisfied in their maternal role. Conversely, a negative experience of childbirth increases women's risk of experiencing health burdens, including a higher incidence of postpartum depression or fear of another birth, which may result in a caesarean section or possibly a refusal to have another pregnancy or birth at all (Urbanová et al. 2021). Women's low satisfaction with childbirth is associated with a higher incidence of posttraumatic stress disorder (PTSD) and depressive symptoms. The prevalence of PTSD after childbirth ranges from 4% to 17% (Grisbrook and Letourneau 2021). Postpartum depression is moderate to severe depression affecting an estimated 13% to 19% of postpartum women (Hutchens and Kearney 2020). This can manifest as mood swings, disinterest in daily activities, fatigue and loss of energy, inability to concentrate, inappropriate worry about the baby, tearfulness, feelings of sadness, decreased sexual libido, disturbed sleep or even thoughts of suicide (Urbanová et al. 2021). Thus, with SSC, women's satisfaction with childbirth and perinatal mental health interact.

## 3 Methods

### 3.1 Aims of the Study

The aims of the study were to: (1) identify perinatal factors associated with the implementation and duration of early maternal‐newborn SSC after birth; (2) examine the association between SSC duration and maternal birth satisfaction assessed with the CZ‐BSS‐R; and (3) examine associations between SSC duration and positive screening for postpartum depression and childbirth‐related posttraumatic stress symptoms.

### 3.2 Design and Data Collection

The study used a retrospective cross‐sectional survey design and was reported in accordance with the STROBE statement. Eligible participants were Czech‐speaking women aged 18 years or older who had given birth within the preceding 24 months. Participants were recruited online using non‐probability convenience sampling. The invitation and the questionnaire were posted in October and November 2022 in six social‐media forums for women on maternity or parental leave in the Czech Republic. This approach enabled recruitment of a large postpartum sample but introduced self‐selection and coverage bias. The survey was administered using Survio, which applies recognized information‐security standards.

### 3.3 Population and Sample

A total of 1318 women responded; 1013 (77%) provided complete questionnaires meeting the inclusion criteria. Multivariate outliers were screened using Mahalanobis distance calculated from the CZ‐BSS‐R items, EPDS and PC‐PTSD‐5 item sets, resulting in a final analytic sample of 985 participants. Because recruitment was non‐probabilistic and internet‐based, the sample should not be interpreted as representative of Czech postpartum women. Only complete questionnaires meeting the inclusion criteria were retained for the analytic workflow; missing questionnaire data were not imputed.

SSC was analysed in four mutually exclusive categories: no SSC, ≤ 30 min, > 30–120 min and > 120 min. An earlier planning estimate based on four groups, 80% power, a two‐sided alpha of 0.05 and a conventional medium effect size of 0.25 indicated a minimum total sample of 180; the final analytic sample exceeded this estimate. This calculation was a planning estimate and was not used to justify the precision of individual regression coefficients.

### 3.4 Measures

#### 3.4.1 Demographics Questionnaire

The demographics questionnaire obtained information regarding age, marital status, education, employment, as well as information regarding parity, number of months since last delivery and health status related to last pregnancy and delivery (course of pregnancy, psychological state before delivery, term of delivery, course of delivery, condition of the child after delivery, etc.). The questionnaire included a question on uninterrupted SSC with the baby after birth (with an explanation for the respondents ‘the baby was on my chest in skin‐to‐skin contact’) and with response options: Yes, for up to 30 min after birth; Yes, for up to 2 h after birth; Yes, for more than 2 h after birth; No, I was not in unhindered skin‐to‐skin contact with the baby after birth. For analysis, these responses were coded as ≤ 30 min, > 30–120 min, > 120 min and no SSC.

#### 3.4.2 The Edinburgh Postnatal Depression Scale

The Edinburgh Postnatal Depression Scale (EPDS; Cox et al. 1987) is a self‐rating scale consisting of ten questions that are rated on a 4‐point scale (minimum score 0 and maximum score 3). Thus, the total score on the EPDS scale can range from 0 to 30, with higher scores indicating more intense symptoms of depression. Postnatal women rate on the scale the severity of depressive symptoms over a period of the last week. A validated Czech‐language version of the EPDS (Břicháček et al. 2000) was used in the current study. The Czech version of the EPDS has good internal consistency (Cronbach's alpha 0.863) and an EPDS score ≥ 11 achieves the best combination of sensitivity and specificity values for detecting major depressive disorder (Horakova et al. 2022).

#### 3.4.3 The Birth Satisfaction Scale‐Revised

The Birth Satisfaction Scale Revised (BSS‐R) was developed by Hollins Martin and Martin (2014) in the UK. The BSS‐R is a valid, reliable instrument to measure women's postpartum satisfaction that can be easily and quickly completed by women and has ten items rated on a 5‐point Likert scale. It has three subscales that measure different domains: quality of care provision (QC), women's personal attributes (WA) and stress experienced during labour (SE). The total score of the BSS‐R ranges between 0 and 40, with higher scores representing higher birth satisfaction. The study used the Czech version of the BSS‐R (CZ‐BSS‐R, Ratislavová et al. 2024), which has good internal consistency (Cronbach's alpha = 0.87).

#### 3.4.4 The Primary Care PTSD Screen for DSM‐5

The Primary Care PTSD Screen for DSM‐5 (PC‐PTSD‐5) is a five‐item questionnaire designed to identify individuals at risk for PTSD. It is not a diagnostic tool. It may be used primarily by primary care health professionals in identifying individuals who may benefit from further assessment and treatment for PTSD symptoms. If an individual has been exposed to a traumatic event, the PC‐PTSD‐5 questionnaire asks five questions (yes, no) about how the event has affected them in the past month. Results from validation studies suggest that a cut‐off of 3 on the PC‐PTSD‐5 questionnaire (respondent answers ‘yes’ to 3 of the 5 questions) is optimally sensitive to probable PTSD in women (Bovin et al. 2021). For our research, we adapted this questionnaire to the situation of childbirth. Thus, the questions were designed, e.g., ‘In the past month, have you had nightmares about childbirth, or have you thought about childbirth without wanting to?’ The adapted Czech version of PC‐PTSD‐5 shows good psychometric properties (Martin et al. 2023).

### 3.5 Data Analysis

Categorical variables are reported as frequencies and percentages, and scale scores as means with standard deviations and medians with interquartile ranges. Participants were classified into four mutually exclusive SSC categories: no SSC, ≤ 30 min, > 30–120 min and > 120 min.

Associations between SSC duration and categorical perinatal characteristics were assessed using Pearson's chi‐square tests with Cramer's V as the effect‐size measure. Differences in CZ‐BSS‐R, EPDS and PC‐PTSD‐5 scores across SSC categories were assessed using Kruskal‐Wallis tests, followed by Dunn's pairwise comparisons with Holm‐Bonferroni adjustment. Epsilon‐squared was reported as the Kruskal‐Wallis effect size.

To address measured confounding, multivariable models treated SSC duration as a categorical exposure with no SSC as the reference category. Linear regression was used for the CZ‐BSS‐R total score. Logistic regression was used for positive screening outcomes defined as EPDS ≥ 11 and PC‐PTSD‐5 ≥ 3. Models adjusted for age, time since birth, parity, pregnancy course, psychological state before birth, mode of birth, birth timing and newborn health status. Adjusted regression coefficients or odds ratios with 95% confidence intervals are reported. Analyses were conducted in R using the prespecified reproducible analysis code. Findings are interpreted as associations rather than causal effects.

### 3.6 Ethics

Ethical approval was gained from the Research Ethics Committee of the University of West Bohemia in Pilsen, reference number ZCU 000065/2023. Participation was voluntary, data were collected anonymously, and electronic informed consent was obtained before questionnaire completion. The study procedures protected participants' dignity, privacy and personal data in accordance with applicable requirements.

## 4 Results

Women were aged 18–44 years (M = 30.76; SD = 4.59). Of the 985 women, 496 were ≤ 12 months postpartum and 489 were > 12 months postpartum (M = 12.49; SD = 7.32). Most participants were married (65.48%), had completed upper‐secondary (31.78%) or university education (52.08%) and were on maternity leave (88.83%; Table 1). Perinatal characteristics by SSC duration are shown in Table 2. SSC categories comprised no SSC (*n* = 339), ≤ 30 min (*n* = 246), > 30–120 min (*n* = 315) and > 120 min (*n* = 85).

**TABLE 1:** Sociodemographic characteristics (*N* = 985).

Variables	*n* (%)
Marital status	
Single	320 (32.49)
Married	645 (65.48)
Divorced	20 (2.03)
Educational status	
Primary school degree	31 (3.15)
Secondary school degree	87 (8.83)
High school degree	313 (31.78)
Higher school degree	41 (4.16)
University degree	513 (52.08)
Employment status	
Employed	80 (8.12)
Unemployed	3 (0.31)
Self‐employed	27 (2.74)
Maternity leave	875 (88.83)

**TABLE 2:** Associations between perinatal characteristics and SSC duration (*N* = 985).

Variable	Category	Total *n* (%)	No SSC *n* (%)	≤ 30 min *n* (%)	> 30–120 min *n* (%)	> 120 min *n* (%)	*χ* ^2^	df	*p*	Cramer's V
Parity	Primipara	648 (65.8)	266 (78.5)	153 (62.2)	189 (60.0)	40 (47.1)	58.19	9	< 0.001	0.140
	Secundipara	275 (27.9)	61 (18.0)	79 (32.1)	103 (32.7)	32 (37.6)				
	Tercipara	46 (4.7)	9 (2.7)	8 (3.3)	21 (6.7)	8 (9.4)				
	Multipara	16 (1.6)	3 (0.9)	6 (2.4)	2 (0.6)	5 (5.9)				
Pregnancy course	Low risk	739 (75.0)	228 (67.3)	184 (74.8)	261 (82.9)	66 (77.6)	39.00	6	< 0.001	0.141
	Risk	188 (19.1)	72 (21.2)	51 (20.7)	47 (14.9)	18 (21.2)				
	Pathological	58 (5.9)	39 (11.5)	11 (4.5)	7 (2.2)	1 (1.2)				
Psychological state before birth	Very good	623 (63.2)	204 (60.2)	161 (65.4)	196 (62.2)	62 (72.9)	15.66	12	0.207	0.073
	Good	176 (17.9)	68 (20.1)	38 (15.4)	58 (18.4)	12 (14.1)				
	Moderate	136 (13.8)	47 (13.9)	29 (11.8)	52 (16.5)	8 (9.4)				
	Bad	35 (3.6)	13 (3.8)	12 (4.9)	8 (2.5)	2 (2.4)				
	Very bad	15 (1.5)	7 (2.1)	6 (2.4)	1 (0.3)	1 (1.2)				
Mode of birth	NSVB	400 (40.6)	52 (15.3)	121 (49.2)	182 (57.8)	45 (52.9)	337.60	18	< 0.001	0.338
	MVB	243 (24.7)	54 (15.9)	71 (28.9)	94 (29.8)	24 (28.2)				
	CS acute	162 (16.4)	136 (40.1)	10 (4.1)	7 (2.2)	9 (10.6)				
	CS planned	62 (6.3)	45 (13.3)	10 (4.1)	5 (1.6)	2 (2.4)				
	VAVB	62 (6.3)	28 (8.3)	13 (5.3)	18 (5.7)	3 (3.5)				
	Revisio/lysis	42 (4.3)	16 (4.7)	16 (6.5)	9 (2.9)	1 (1.2)				
	Other	14 (1.4)	8 (2.4)	5 (2.0)	0 (0.0)	1 (1.2)				
Birth timing	At term	825 (83.8)	246 (72.6)	211 (85.8)	292 (92.7)	76 (89.4)	61.51	6	< 0.001	0.177
	Premature < 37 g.w.	95 (9.6)	63 (18.6)	20 (8.1)	10 (3.2)	2 (2.4)				
	Post‐term > 42 g.w.	65 (6.6)	30 (8.8)	15 (6.1)	13 (4.1)	7 (8.2)				
Newborn health status	Healthy	852 (86.5)	257 (75.8)	217 (88.2)	299 (94.9)	79 (92.9)	55.92	3	< 0.001	0.238
	Health problems	133 (13.5)	82 (24.2)	29 (11.8)	16 (5.1)	6 (7.1)				
Time since birth	≤ 12 months	496 (50.4)	144 (42.5)	121 (49.2)	179 (56.8)	52 (61.2)	17.81	3	< 0.001	0.134
	> 12 months	489 (49.6)	195 (57.5)	125 (50.8)	136 (43.2)	33 (38.8)				

*Note:* Percentages in SSC columns are column percentages and describe the composition of each SSC‐duration group; they should not be interpreted as probabilities of receiving SSC within a perinatal category. Pearson's chi‐square test was used; Cramer's V is reported as effect size. Statistical significance was set at *p*‐value < 0.05. Associations involving sparse categories should be interpreted cautiously.

Abbreviations: CS, caesarean section; MVB, medicated vaginal birth; NSVB, normal spontaneous vaginal birth; SSC, skin‐to‐skin contact; VAVB, vacuum‐assisted vaginal birth.

SSC duration was associated with several perinatal characteristics. The strongest association was observed for mode of birth (*χ* ^2^ = 337.60, df = 18, *p* < 0.001, Cramer's V = 0.338). SSC also differed by parity (*χ* ^2^ = 58.19, df = 9, *p* < 0.001, Cramer's V = 0.140), pregnancy course (*χ* ^2^ = 39.00, df = 6, *p* < 0.001, Cramer's V = 0.141), birth timing (*χ* ^2^ = 61.51, df = 6, *p* < 0.001, Cramer's V = 0.177), newborn health status (*χ* ^2^ = 55.92, df = 3, *p* < 0.001, Cramer's V = 0.238) and time since birth greater than 12 months (*χ* ^2^ = 17.81, df = 3, *p* < 0.001, Cramer's V = 0.134). Psychological state before birth was not significantly associated with SSC duration in the unadjusted chi‐square analysis (*χ* ^2^ = 15.66, df = 12, *p* = 0.207, Cramer's V = 0.073).

Inspection of the column percentages in Table 2 showed that the no‐SSC group contained a larger proportion of primiparous women (78.5%) than the SSC‐duration groups (47.1%–62.2%). The no‐SSC group also contained higher proportions of pathological pregnancies (11.5%), acute and planned caesarean births (40.1% and 13.3%, respectively), preterm births (18.6%) and newborn health problems (24.2%). By contrast, spontaneous vaginal birth accounted for 49.2%–57.8% of each SSC‐duration group but only 15.3% of the no‐SSC group. Women more than 12 months postpartum constituted 57.5% of the no‐SSC group, compared with 43.2% of the > 30–120‐min group and 38.8% of the > 120‐min group. These percentages describe the composition of the SSC‐duration groups and should not be interpreted as probabilities of receiving SSC within each perinatal category.

Maternal wellbeing scale scores by SSC duration are shown in Table 3. Women reporting no SSC had the lowest CZ‐BSS‐R scores and the highest EPDS and PC‐PTSD‐5 scores. Kruskal–Wallis tests showed differences across SSC categories for CZ‐BSS‐R (*χ* ^2^ = 158.68, df = 3, *p* < 0.001, *ε* ^2^ = 0.159), EPDS (*χ* ^2^ = 44.07, df = 3, *p* < 0.001, *ε* ^2^ = 0.042) and PC‐PTSD‐5 (*χ* ^2^ = 73.88, df = 3, *p* < 0.001, *ε* ^2^ = 0.072). Dunn's pairwise comparisons were interpreted using Holm‐Bonferroni‐adjusted *p*‐values.

**TABLE 3:** Maternal wellbeing scale scores according to SSC duration.

Scale	SSC duration	M (SD)	Median [IQR]	Kruskal–Wallis *χ* ^2^	df	*p*	*ε* ^2^
CZ‐BSS‐R	No SSC	18.46 (8.27)	18.0 [12.0–25.0]	158.68	3	< 0.001	0.159
CZ‐BSS‐R	≤ 30 min	24.02 (8.43)	25.0 [18.0–31.0]				
CZ‐BSS‐R	> 30–120 min	25.96 (8.15)	28.0 [21.0–32.0]				
CZ‐BSS‐R	> 120 min	28.86 (7.93)	29.0 [23.0–36.0]				
EPDS	No SSC	11.15 (7.00)	10.0 [6.0–16.0]	44.07	3	< 0.001	0.042
EPDS	≤ 30 min	9.57 (6.34)	9.0 [5.0–14.0]				
EPDS	> 30–120 min	8.01 (6.15)	7.0 [3.0–12.0]				
EPDS	> 120 min	7.42 (5.58)	6.0 [3.0–11.0]				
PC‐PTSD‐5	No SSC	1.92 (1.57)	2.0 [1.0–3.0]	73.88	3	< 0.001	0.072
PC‐PTSD‐5	≤ 30 min	1.30 (1.36)	1.0 [0.0–2.0]				
PC‐PTSD‐5	> 30–120 min	1.02 (1.19)	1.0 [0.0–2.0]				
PC‐PTSD‐5	> 120 min	0.94 (1.29)	0.0 [0.0–1.0]				

*Note:* SSC categories are mutually exclusive: ≤ 30 min, > 30–120 min, > 120 min and no SSC. Kruskal–Wallis tests were followed by Dunn's post hoc tests with Holm‐Bonferroni correction for multiple comparisons. Statistical significance was set at adjusted *p*‐value < 0.05.

Abbreviations: CZ‐BSS‐R, Czech version of the Birth Satisfaction Scale‐Revised; EPDS, Edinburgh Postnatal Depression Scale; PC‐PTSD‐5, Primary Care PTSD Screen for DSM‐5; SSC, skin‐to‐skin contact.

Values indicative of increased risk of postpartum depression (EPDS ≥ 11) occurred in 386 respondents (39.19%). The proportion was highest among women without SSC (169/339, 49.85%), followed by ≤ 30 min of SSC (100/246, 40.65%), > 30–120 min of SSC (94/315, 29.84%) and > 120 min of SSC (23/85, 27.06%). Values indicative of increased risk of posttraumatic stress symptoms (PC‐PTSD‐5 ≥ 3) occurred in 206 respondents (20.91%). The proportion was also highest among women without SSC (111/339, 32.74%), followed by ≤ 30 min of SSC (49/246, 19.92%), > 30–120 min of SSC (37/315, 11.75%) and > 120 min of SSC (9/85, 10.59%).

Adjusted associations are shown in Table 4. Compared with no SSC, adjusted CZ‐BSS‐R scores were higher for SSC ≤ 30 min (B = 2.31, 95% CI 0.91–3.70, *p* = 0.001), > 30–120 min (B = 3.30, 95% CI 1.89–4.70, *p* < 0.001) and > 120 min (B = 6.10, 95% CI 4.18–8.02, *p* < 0.001).

**TABLE 4:** Adjusted associations of SSC duration with maternal wellbeing outcomes.

Outcome	Model/effect	SSC contrast	Estimate	95% CI (LCL‐UCL)	*p*
CZ‐BSS‐R total score	Linear regression B	≤ 30 min vs. No SSC	2.31	0.91–3.70	0.001
CZ‐BSS‐R total score	Linear regression B	> 30–120 min vs. No SSC	3.30	1.89–4.70	< 0.001
CZ‐BSS‐R total score	Linear regression B	> 120 min vs. No SSC	6.10	4.18–8.02	< 0.001
EPDS ≥ 11	Logistic regression aOR	≤ 30 min vs. No SSC	0.80	0.53–1.20	0.282
EPDS ≥ 11	Logistic regression aOR	> 30–120 min vs. No SSC	0.50	0.33–0.76	0.001
EPDS ≥ 11	Logistic regression aOR	> 120 min vs. No SSC	0.46	0.26–0.84	0.011
PC‐PTSD‐5 ≥ 3	Logistic regression aOR	≤ 30 min vs. No SSC	0.72	0.45–1.16	0.173
PC‐PTSD‐5 ≥ 3	Logistic regression aOR	> 30–120 min vs. No SSC	0.45	0.27–0.75	0.003
PC‐PTSD‐5 ≥ 3	Logistic regression aOR	> 120 min vs. No SSC	0.35	0.15–0.78	0.010

*Note:* No SSC was the reference category. Models were adjusted for age, time since birth in months, parity, pregnancy course, psychological state before birth, mode of birth, birth timing and newborn health status. Statistical significance was set at *p*‐value < 0.05. Estimates represent associations and not causal effects.

Abbreviations: aOR, adjusted odds ratio; B, adjusted unstandardised regression coefficient; CI, confidence interval; CZ‐BSS‐R, Czech version of the Birth Satisfaction Scale‐Revised; EPDS, Edinburgh Postnatal Depression Scale; LCL, lower confidence limit; PC‐PTSD‐5, Primary Care PTSD Screen for DSM‐5; SSC, skin‐to‐skin contact; UCL, upper confidence limit.

For EPDS ≥ 11, adjusted odds were lower for SSC > 30–120 min (aOR = 0.50, 95% CI 0.33–0.76, *p* = 0.001) and > 120 min (aOR = 0.46, 95% CI 0.26–0.84, *p* = 0.011), but not for SSC ≤ 30 min (aOR = 0.80, 95% CI 0.53–1.20, *p* = 0.282). For PC‐PTSD‐5 ≥ 3, adjusted odds were lower for SSC > 30–120 min (aOR = 0.45, 95% CI 0.27–0.75, *p* = 0.003) and > 120 min (aOR = 0.35, 95% CI 0.15–0.78, *p* = 0.010), but not for SSC ≤ 30 min (aOR = 0.72, 95% CI 0.45–1.16, *p* = 0.173).

## 5 Discussion

This study examined perinatal factors associated with early maternal‐newborn SSC and associations between SSC duration, birth satisfaction and postpartum mental wellbeing. SSC duration differed most strongly by mode of birth and was also associated with parity, pregnancy course, birth timing, newborn health status and time since birth. It is recommended that all clinically stable, viable newborns born vaginally or by caesarean section should be in immediate SSC for at least the first hour of life or until the first breastfeeding is completed (Brimdyr et al. 2023).

In our sample, a high proportion of women reported no SSC, suggesting that implementation of this evidence‐based practice may be inconsistent. However, because the study used retrospective self‐report and non‐probability sampling, these findings should be interpreted as indicators of potential practice variation rather than population prevalence estimates. Research conducted in the Czech Republic (Begley et al. 2018) yielded the finding that in many places of obstetric care there is still a paternalistic approach to women in labour, with frequent elements of routine care that are not based on evidence‐based practice (EBP). Many obstetric practices are not only not evidence‐based but are non‐consensual and abusive to women. Such practices can leave women with lifelong physical and emotional trauma (Begley et al. 2018). Another large‐scale study that investigated women's own experience of perinatal care in the Czech Republic (Wilhelmová et al. 2025) found that care provided by obstetric staff is not individualized with sufficient time and space to support women, especially in the postpartum period and also reported limited support for SSC during caesarean delivery. In September 2024, the government approved a comprehensive strategy for the Baby‐friendly Hospital Initiative, which aims to improve care for women and newborns in Czech maternity hospitals. The measures in the new strategy, to be implemented by 2030, also include uninterrupted contact between mother and child after birth.

Our results show that women with acute and elective caesarean section reported less frequent SSC after delivery than women with spontaneous vaginal delivery. The goal of all health care providers providing obstetric care should be to offer all women the same quality of EBP care, which includes SSC after caesarean delivery (Junk‐Wilson et al. 2024). SSC contact in women after or during caesarean section has many benefits (support for lactation, uterine involution, maternal role adaptation and psychological wellbeing). Women often express feelings of helplessness and dependency after caesarean section. SSC can provide an opportunity for these women to re‐engage in the process and support their maternal competence (Brubaker et al. 2019).

Women who had given birth more than 12 months before completing the survey more often reported no SSC than those who had given birth within the preceding 12 months. Pandemic‐related maternity‐care restrictions may have contributed to this temporal pattern (Janoušková and Matulníková 2023). However, because the study did not collect data on hospital policies or participants' individual exposure to such restrictions, the finding cannot be attributed to the COVID‐19 pandemic and should be interpreted cautiously.

Another objective was to examine the association between women's satisfaction at birth, assessed by the CZ‐BSS‐R and early SSC. Our results indicate that absence of SSC was associated with lower birth satisfaction, while longer SSC duration was associated with higher birth satisfaction. This pattern remained after adjustment for selected perinatal and maternal factors. Similar associations between SSC and birth satisfaction have been reported in previous studies (Brubaker et al. 2019; Kahalon et al. 2021; Mazúchová et al. 2020).

The importance of SSC for maternal mental health is evidenced in a number of studies (Kirca and Adibelli 2021). These include research that confirms the positive effect of daily SSC in the first weeks postpartum on reducing maternal depressive symptoms and physiological stress in the postpartum period, on preventing the increase in anxiety and reducing symptoms of fatigue in the first 12 weeks postpartum (Cooijmans et al. 2022). Early SSC initiates emotional bonding between mother and child and triggers hormonal and psychological processes in the woman's body, also significantly promoting lactation development. During SSC, the maternal oxytocinergic system is activated via nerve fibres upon touch (Cooijmans et al. 2022). Oxytocin modulates social interactive behaviour, maternal behaviour, bonding and attachment formation between mother and infant. Increased concentrations of oxytocin may be able to reduce the activity of the hypothalamic–pituitary–adrenal axis and the sympathetic nervous system, thereby reducing the body's physiological response to fear and stress. A number of studies have reported a significant inverse relationship between higher perinatal oxytocin levels and maternal symptoms of postpartum depression (Scime et al. 2019). Higher oxytocin levels are associated with improved maternal mental health, including reduced symptoms of stress and anxiety. SSC leads to an increase in maternal oxytocin levels and a decrease in cortisol levels. Uvnäs Moberg et al. (2022) reported that social interaction skills as well as improved ability to cope with stressful situations were observed in women up to 1 year after SSC was performed immediately after delivery. Manifestations of improved mother‐infant interaction and increased stress coping skills were only found when the mother and child were allowed to undertake SSC immediately after delivery. No lasting effects were found when SSC was provided 2 h later (Bystrova et al. 2009). Immediately after birth, the level of oxytocin, catecholamines and dopamine is very high. Such high levels are apparently not reached under any other circumstances during life, out of the normal range (Olza et al. 2020). In addition to promoting dopamine release, these high levels of oxytocin may also cause activation of serotonin pathways and vice versa. Women experience euphoria, oxytocin has an effect on attenuated pain reflex, activates the brain's reward centers, sensitizes the mother's skin and affects emotional memory (Buckley et al. 2023). All of these may have long‐term effects on the maternal psyche after the birth. In the present study, SSC durations longer than 30 min were associated with lower adjusted odds of positive screening for postpartum depression and childbirth‐related posttraumatic stress symptoms, whereas SSC ≤ 30 min was not statistically significant after adjustment. These biological and relational mechanisms were not measured and should therefore be interpreted as plausible hypotheses rather than demonstrated mediators.

For nursing practice, these findings support continued attention to SSC as a feasible component of immediate postpartum and neonatal care, especially in situations where SSC is often interrupted, such as caesarean section, preterm birth or newborn health complications.

## 6 Strengths and Limitations

Several limitations of this study need to be addressed. First, the sample was obtained online. The advantage of online research is obtaining a large sample of women in a short period of time. However, the sample is then limited to those respondents who have access to social media and technology. Also, women who were more likely to participate in the research were more likely to be preoccupied with the topic, more psychologically affected by childbirth and more traumatized. This is evidenced by the higher percentage of women with postpartum depression and PTSD than reported in other studies. We recognize that relying solely on internet‐mediated recruitment may have introduced selection bias by under‐representing women with limited online access or lower digital literacy—such as those from socioeconomically disadvantaged or rural communities—who might exhibit different prevalence and risk profiles. Digitally engaged participants may also possess greater health literacy or motivation to seek support, potentially inflating symptom estimates and affecting observed associations. To bolster external validity, future studies should employ a hybrid recruitment strategy, combining online platforms with community‐ and clinic‐based enrolment to capture a more demographically and socioeconomically diverse cohort.

Second, the retrospective cross‐sectional design precludes conclusions about temporality, direction or causality and is vulnerable to recall bias. Third, although the models adjusted for measured maternal, pregnancy, birth and newborn factors, residual confounding is likely; hospital policies, detailed neonatal condition, previous mental‐health diagnoses, social support, breastfeeding support and quality of intrapartum care were not fully measured. Fourth, the adapted childbirth‐related PC‐PTSD‐5 is a screening measure rather than a diagnostic assessment. Fifth, multiple outcomes and comparisons increase the risk of chance findings despite Holm‐Bonferroni adjustment for post hoc tests. Sixth, Mahalanobis‐based exclusion may affect generalisability and should be considered when comparing the results with studies using unfiltered samples. Seventh, no population weights were available or applied; consequently, the findings should not be interpreted as population‐representative estimates. Blinding was not applicable because exposure and outcomes were retrospectively self‐reported. Replication using prospective designs, representative sampling and hospital‐level data is needed across diverse settings.

## 7 Conclusion

Each birth greatly affects the overall health of the woman and the newborn. Healthcare professionals who care for the woman and baby during childbirth should provide highly skilled but also sensitive and empathetic care. An essential component of care after physiological birth should be timely and uninterrupted SSC when clinically feasible and safe.

In this retrospective cross‐sectional sample, all SSC‐duration groups were associated with higher adjusted birth satisfaction than no SSC, while SSC durations exceeding 30 min were associated with lower adjusted odds of positive screening for postpartum depression and childbirth‐related posttraumatic stress symptoms. These associations should not be interpreted as causal effects.

In conclusion, our findings emphasize the need for education and support about early SSC (especially after caesarean section and after preterm birth) for both health professionals and pregnant persons. Clinical practice guidelines for midwives, obstetric and neonatal nurses should focus not only on initiating SSC but also on sustaining its duration and safely overcoming barriers during complicated births. By addressing organizational and clinical barriers, midwives, neonatal nurses, obstetric nurses and obstetricians may contribute to more consistent, safe and woman‐centred implementation of SSC.

## Author Contributions

**Eva Hendrych Lorenzová:** conceptualization, methodology, investigation, writing – original draft, writing – review and editing, resources. **Denis Mainz:** methodology, formal analysis, funding acquisition, writing – original draft, writing – review and editing, data curation. **Alena Lochmannová:** methodology, writing – original draft, writing – review and editing, resources. **Colin R. Martin:** conceptualization, methodology, data curation, formal analysis, writing – original draft, writing – review and editing. **Kateřina Ratislavová:** conceptualization, methodology, investigation, supervision, project administration, writing – original draft, writing – review and editing.

## Funding

This research was funded by the Institutional Support for the Long‐term Conceptual Development of a Research Organization, provided by the Ministry of Education, Youth and Sports of the Czech Republic. The Article Publication Charge for this article was funded by the Propolis 33 Foundation. The Ministry of Education, Youth and Sports of the Czech Republic had no role in the study design; data collection, analysis or interpretation; preparation of the manuscript; or the decision to submit the article for publication. The Propolis 33 Foundation provided financial support solely for the Article Publication Charge after acceptance and had no role in the study design or conduct; data collection, analysis or interpretation; preparation of the manuscript; peer‐review process; or editorial decision.

## Disclosure

OpenAI Codex and Anthropic Claude were used solely as technical support tools to assist with testing, debugging and optimizing the syntax of author‐developed R code implementing the statistical procedures specified by the authors and with technical consistency checking and formatting of statistical outputs. Grammarly AI and DeepL were used for language editing and proofreading; the accuracy and appropriateness of scientific and clinical terminology were reviewed by the authors. The AI tools did not generate the source data and did not independently determine the study design, statistical methodology, scientific interpretation or conclusions. All AI‐assisted outputs incorporated into the manuscript, as well as all scientific content, analyses, interpretations, conclusions and the final manuscript text, were critically reviewed, verified and approved by all authors. AI tools were not listed as authors and did not assume responsibility for any part of the work. The authors retain full responsibility for the accuracy, integrity and final content of the manuscript.

Patient or Public Contribution: There was no patient or public involvement in the design, conduct, analysis or preparation of the manuscript; postpartum women participated as consenting survey respondents.

Statistics: The authors have checked that the submission conforms, as applicable, to the journal's statistical guidelines. The author team includes statisticians: Denis Mainz, PhD, e‐mail: dmainz@fzs.zcu.cz and prof. Colin R. Martin, e‐mail: colinmartin.bssr@proton.me. The authors affirm that the analytical methods are suitably applied to the data within the study design and context and that the statistical findings have been implemented and interpreted correctly. The authors accept responsibility for the appropriateness, conduct and interpretation of the statistical approach.

## Ethics Statement

Ethical approval was gained from The Research Ethics Committee of the University of West Bohemia in Pilsen, reference number ZCU 000065/2023. The Research Ethics Committee's decision confirmed that the study participants are volunteers, their human dignity is not violated and they have not been exposed to physical, psychological or social risks. Only anonymous data were collected from the study participants and their privacy and personal data protection is guaranteed according to the relevant law. Electronic informed consent was obtained before questionnaire completion.

## Conflicts of Interest

The authors declare no conflicts of interest.

## Data Availability

The data that support the findings of this study are available from the corresponding author upon reasonable request.
